# Analysis of the Effect of the Tablet Matrix on the Polymorphism of Ibuprofen, Naproxen, and Naproxen Sodium in Commercially Available Pharmaceutical Formulations

**DOI:** 10.3390/mps8050099

**Published:** 2025-09-01

**Authors:** Edyta Leyk, Marcin Środa, Gracjan Maślanka, Patrycja Nowaczyk, Amelia Orzołek, Hanna Grodzka, Aleksandra Kurek, Olaf Knut, Julia Michalak, Jonatan Płachciak, Alina Plenis

**Affiliations:** 1 Department of Analytical Chemistry, Faculty of Pharmacy, Medical University of Gdansk, Gen. J. Hallera 107, 80-416 Gdansk, Poland; edyta.leyk@gumed.edu.pl (E.L.); gmaslanka@gumed.edu.pl (G.M.); p.nowaczyk@gumed.edu.pl (P.N.); ameliaorzolek@gumed.edu.pl (A.O.); hannagrodzka@gumed.edu.pl (H.G.); aleksandra.kurek@gumed.edu.pl (A.K.); olfinx@gumed.edu.pl (O.K.); juliamichalak1234567@gumed.edu.pl (J.M.); jonatan.plachciak@gumed.edu.pl (J.P.); 2 Faculty of Materials Science and Ceramics, AGH University of Science and Technology, A. Mickiewicza 30, 30-059 Kraków, Poland; msroda@agh.edu.pl

**Keywords:** ibuprofen, naproxen, naproxen sodium, pharmaceutical formulations, polymorphism, DSC, FTIR, Raman, XRD

## Abstract

Pharmaceutical formulations, in addition to the medicinal substance(s), contain added excipients that make it possible to create a pharmaceutical product that exhibits required properties in terms of mechanical, physical, chemical, and microbiological stability. Additionally, these substances can act as release modifiers or improve bioavailability parameters. Literature data indicate that excipients, especially polymeric ones, can also affect the polymorphism of the active substance, resulting in drug bioavailability enhancement or reduction. This influence can be evaluated using thermal and spectroscopic methods. In the study, differential scanning calorimetry (DSC), vibrational spectroscopic studies (Fourier transform infrared spectroscopy, FTIR), Raman spectroscopy, and X-ray diffraction (XRD) assay of ibuprofen, naproxen, and naproxen sodium standards and pharmaceutical preparations containing these medicinal substances in their compositions were carried out. DSC results indicated that a sharp melting peak was observed on the DSC curves of the standards, confirming their crystalline form. DSC results obtained for pharmaceutical formulations also indicated that the enthalpy of melting is sometimes lower than calculated from the percentage of active ingredients in the formulations. In addition, the melting peak is often broadened and shifted toward lower temperatures, suggesting the influence of excipients on the polymorphism of drug substances. The FTIR and Raman spectra of pharmaceutical formulations contained all characteristics of the active substances. XRD analysis was also performed. Therefore, possible chemical interactions between the components of the preparations have been excluded. At the same time, FTIR and Raman spectroscopy results as well as XRD assay showed a reduction in the height of signals corresponding to the crystalline API form, confirming the possibility of reducing API crystallinity in pharmaceutical formulations.

## 1. Introduction

Pharmaceutical products, apart from the active pharmaceutical ingredient (API) or APIs, also contain excipients [1,2,3,4]. The appropriate selection of excipients may be related to the effect on API polymorphism and, at the same time, on bioavailability.

As excipients in solid formulations, substances acting as diluents, binders, disintegrants, lubricants, and sometimes colorants are mainly used [4,5,6]. Cellulose derivatives (hypromellose, methylcellulose, ethylcellulose, microcrystalline cellulose), sugars (lactose, sucrose), and phosphates (dibasic calcium phosphate) are mainly used as diluents. Their role is to give the product the right volume and mass, especially when the amount of API is small. Starch derivatives (pregelanized starch), synthetic polymers (polyvinylpyrrolidone, PVP), or microcrystalline cellulose are used as binders. The role of these ingredients is to physically integrate the components of the solid formulation after product administration. The role of disintegrants is to efficiently dissolve the formulation after administration, ensuring that the API is released within a sufficiently short time after administration. Starch derivatives (starch carboxymethyl ether or sodium starch carboxymethyl ether) and cross-linked polymers (crospovidone, croscarmellose sodium) are used as binding agents. During the development of solid formulations, lubricants are also added, most commonly stearic acid or magnesium stearate. The purpose of these ingredients is to ensure that after the tableting process, the finished tablet is easily removed from the tablet matrix without damaging the tablet press.

A significant limitation in achieving the desired therapeutic effect is bioavailability, which refers to the percentage of the dose that penetrates the bloodstream after product administration [7,8]. For many APIs, low bioavailability is a serious limitation in pharmacotherapy and necessitates the administration of higher doses to achieve a therapeutic effect. Numerous reports in the literature indicate that substances used as excipients may influence API polymorphism [9,10,11,12]. Research into this effect is of great importance, as a change in polymorphic form, partial or complete amorphization, usually leads to improved bioavailability. An API in amorphous form usually exhibits better bioavailability compared to the most stable crystalline form used in pharmaceutical production.

Most often, the effect of excipients on APIs involves obtaining an amorphous form after co-melting the API with a polymeric excipient [13,14,15]. One of the processes used to obtain melted mixtures of APIs and excipients is hot-melt extrusion (HME). The advantage of this solution is that there is no need to add solvents during the process [12,16]. Obtaining an amorphous form using traditional methods requires the use of solvents, which often have a negative impact on the environment.

In many cases, partial reduction of API crystallinity is also possible without melting, after homogenization of the components in the solid phase [15,17,18,19]. Such interactions are particularly interesting because the preparation of a powder mixture for tableting usually involves the creation of a physical mixture of solid ingredients by mixing them for many hours. Sometimes, due to the properties of the powder mixture, it is necessary to carry out an additional granulation process [20,21]. In this process, small powder particles are bonded together into agglomerates. This procedure requires the addition of a binder, which is usually an aqueous polymer solution. The material must then be dried, which requires exposing the granules to high temperatures. Water and elevated temperatures can further initiate transformations leading to polymorphic changes or reduced crystallinity. The stage of pharmaceutical product manufacturing in which polymorphic transformations may also occur is tableting [9]. During this process, the product is subjected to high pressure, which increases the likelihood of polymorphic transformations. Research into the impact of individual excipients on API polymorphism is very important because it helps to understand the nature of these interactions and to better select excipients for APIs at the product design stage. However, due to the specificity of the tablet matrix and the possible influence of the manufacturing process, it is necessary to monitor API polymorphism in the final pharmaceutical product.

The literature provides evidence that the reduction in ibuprofen crystallinity may occur after homogenization with cross-linked polyvinylpyrrolidone (PVP-CL) [16]. A fully amorphous form can be obtained by co-grinding ibuprofen with kaolin [22] or in the HME process after co-melting ibuprofen with hydroxypropylmethylcellulose (HPMC) E5 or Eudragit^®^ E PO [23,24].

The reduction in the crystallinity of naproxen can be achieved by preparing a physical mixture with HPMC, and after melting this mixture, naproxen can be completely amorphized [19]. The reduction in crystallinity of naproxen and naproxen sodium can be achieved by spray drying a mixture of APIs with HPMC [25]. Total amorphization of naproxen is also possible after co-melting with polyvinylpyrrolidone (PVP) [26].

The most-used methods in API and pharmaceutical product testing are gas chromatography (GC), liquid chromatography (LC), and mass spectrometry (MS) [27]. These GC, LC, and MS methods are designed for molecular separation, but they do not allow for the analysis of polymorphic forms. Methods commonly used to study polymorphic and amorphous forms include differential scanning calorimetry (DSC), X-ray diffraction (XRD), and spectroscopy methods in infrared range, most commonly Fourier transform infrared (FTIR), near infrared (NIR) and Raman spectroscopy [21,28,29]. DSC curves enable the assessment of polymorphic forms based on the melting point of the crystalline form, as this value is characteristic and varies depending on the polymorphic form of the substance. In quantitative analysis, the characteristic value in DSC measurements is the heat of melting of the crystalline form. In FTIR, Raman spectroscopy studies how the spectra of individual polymorphic and amorphous forms differ slightly in the position of one or more bands. Similarly, in XRD pattern, a reduction in the height of peaks indicating crystalline form was observed. Sometimes these differences only concern changes in the proportions of the signals.

## 2. Materials and Methods

### 2.1. Materials

References substances: ibuprofen (2-[4-(2-methylpropyl)phenyl]propanoic acid, LOT: R5Y8G-DG) was obtained from Tokyo Chemical Industry Co. (Toshima, Tokyo, Japan). Naproxen ((S)-(+)-6-Methoxy-α-methyl-2-naphthaleneacetic acid, LOT: SLBV6889), naproxen sodium ((S)-6-Methoxy-α-methyl-2-naphthaleneacetic acid sodium salt, LOT: MKCR5501), and paracetamol (acetaminophen, 4-hydroxyacetanilide, 4-acetamonophenol, LOT: SLBH0185V)) were purchased from Sigma-Aldrich (St. Louis, MI, USA). References were obtained as solid substances and used without additional preparation.

A total of 37 pharmaceutical solid formulations, available in Polish pharmacies, containing ibuprofen (25 products), naproxen (6 products), and naproxen sodium (6 products) as the active ingredients (Supplementary Material, Table S1), were used. In this group:

- Fourteen products (Ibumax, Ibupar forte, Ibuprex Max, Ibuprofen Aflofarm, Ibuprofen TZF, Ibuprom, Ibuprom Max, Ibuprom RR MAX, Ibuprom Ultramax, Iburapid, Ibuprofen Max PolfaŁódź, MIG, Nurofen, Nurofen Forte) contained ibuprofen as the only active ingredient.
- Eight products (Acatar Zatoki, Ibum Zatoki Max, Ibuprom Zatoki, Ibuprom Zatoki Max, Infex Zatoki, Metafen Zatoki, Modafen Extra Grip, Nurofen Zatoki) contained ibuprofen and pseudoephedrine hydrochloride as the second active ingredient.
- One product (Ibuprom Zatoki Tabs) contained ibuprofen and phenylephrine hydrochloride as the second active ingredient.
- Two products (APAP intense, Metafen) contained ibuprofen and paracetamol as the second active ingredient.
- Six products contained naproxen (Anapran EC, Apo Napro 250, Apo Napro 500, Naproxen Hasco, Naproxen Hasco 500, Naproxen Polfarmex).
- Six products contained naproxen sodium (Aleve, Anapran, Nalgesin, Nalgesin Forte, Nalgesin Mini, Naxii).

Pharmaceutical solid formulations were crushed with a pestle in a mortar to obtain powdered samples for testing. The powdered samples were not ground to avoid initiating phase changes.

### 2.2. Differential Scanning Calorimetry

Thermal tests were conducted using a heat-flux DSC 822e Mettler Toledo (Schwerzenbach, Switzerland) device. The instrument was cooled with liquid nitrogen, and the analysis was conducted in an atmosphere of nitrogen (purity 99.9997%, Air Products, Warsaw, Poland) flowing at a rate of 70 mL/min. Then, 3.90–4.10 mg of the sample was weighed into an aluminum crucible with a pin using a Mettler Toledo XA105 balance (Schwerzenbach, Switzerland). The crucibles were pressed with a lid in which two symmetrical holes had been made. An empty crucible with a lid was used as a reference. Samples containing ibuprofen or naproxen were heated in the range of 25–250 °C, while samples containing naproxen sodium were heated in the range of 25–300 °C. The heating rate was 10 °C/min, regardless of the active substance contained in the sample. The instrument was controlled and the DSC curves were analyzed using STARe 15.0 software. The DSC device was calibrated using indium (In, purity 99.999%) and zinc (Zn, purity 99.998%).

### 2.3. Spectroscopic Methods

Spectra in the mid-infrared range (4000–400 cm^−1^) were obtained using a Thermo Fisher Scientific Nicolet 380 FTIR spectrometer (Madison, WI, USA), controlled by OMNIC software. The device is equipped with a deuterated triglycine sulfate (DTGS) detector with a KBr window. Then, 1 mg of the sample was homogenized with 100 mg of KBr (spectroscopy grade, Merck, Darmstadt, Germany) in an agate mortar and compressed into a pellet in a hydraulic press (Specac, Orpington, UK). The spectrum was recorded 16 times with a resolution of 2 cm^−1^. The background spectrum was recorded before each measurement.

A Thermo Fisher Scientific DXR SmartRaman spectrometer (Madison, WI, USA) was used in Raman spectroscopy. The spectrometer was equipped with a 15-mW DXR 780 nm laser (aperture of 25 µm), a Raleigh filter, and a charge-coupled detector (CCD). The OMNIC software version 8.2 controlled the instrument and was used for spectral analysis. The spectra were recorded twice in the range of 3413–99 cm^−1^ with a spectral resolution of 2 cm^−1^.

### 2.4. X-Ray Diffraction

XRD measurements of active substances and powdered pharmaceutical products were performed using an HZG-4 device equipped with a CuKα copper lamp in the measurement range of 5–45 (degrees) 2theta. The measurements were conducted with a scanning rate of 4°/min and a step size of 0.05 (degrees) 2theta.

## 3. Results

### 3.1. Ibuprofen Products

#### 3.1.1. DSC Results

**Figure 1:**
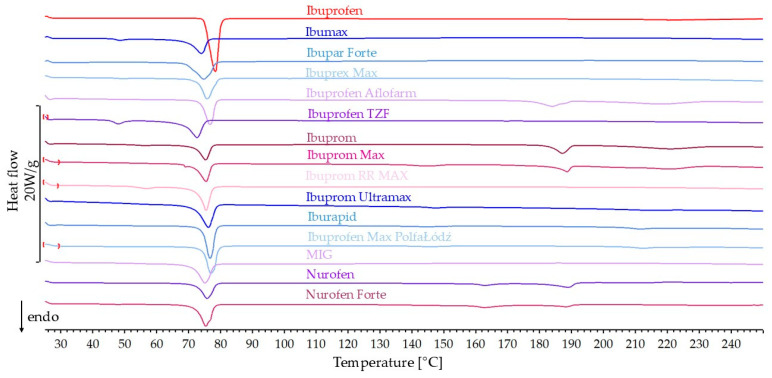
DSC curves of ibuprofen standard and pharmaceutical products with ibuprofen.

An analysis of the DSC curves of the ibuprofen standard and products containing this active substance was performed. The DSC curves of ibuprofen and products containing ibuprofen as the only active ingredient are shown in Figure 1. Table 1 presents the values of heat of melting (J/g), the temperature at which the phase transition process begins (onset), and peak temperature (peak) obtained during the DSC analysis of the samples.

The ibuprofen standard DSC curve shows a sharp melting peak beginning at 74.7 °C. On the DSC curves of all pharmaceutical products containing ibuprofen as the only active substance, the onset of ibuprofen melting was observed in the range of 68.1–74.7 °C. Only in the case of Ibuprofen Max PolfaŁódź is this peak consistent with the melting point of the reference ibuprofen. For the other products, the phase transition began at lower temperatures than for the standard ibuprofen.

Based on the weight of individual dosage units (average of three units) and the declared API content in the pharmaceutical product, the percentage API content in the product was calculated. These values for the tested pharmaceutical products are presented in Table 1. The API content in products with ibuprofen as the only active ingredient is within the range of 39.4–77.3%. Based on this content, the predicted heat of melting for ibuprofen contained in the product was calculated. Next, the predicted and measured heat of melting values were used. The percentage of the predicted value corresponding to the measured value was calculated and is presented in Table 1. The measured values for most ibuprofen products are within the range of 71.6–92.6% of the predicted. The exceptions are two products for which these values are higher than expected by 108.8 and 116.6% for Ibuprofen Aflofarm and Nurofen Forte, respectively. On the DSC curves of several products, in addition to the melting peak of ibuprofen, additional small, strongly broadened signals from excipients were observed.

**Figure 2:**
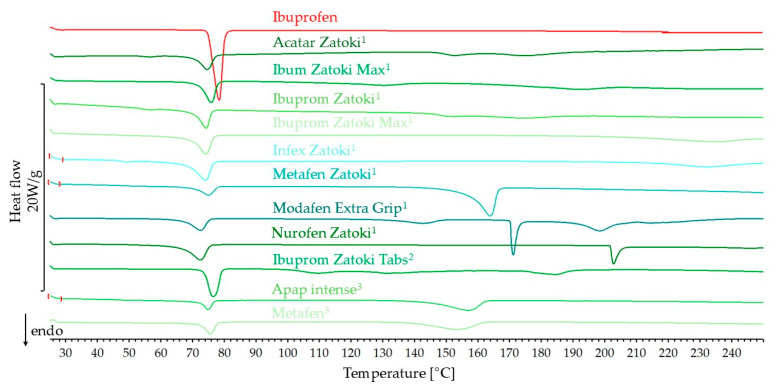
DSC curves of ibuprofen standard, paracetamol standard, and pharmaceutical products with ibuprofen and with the second API (^1^ pseudoephedrine hydrochloride; ^2^ phenylephrine hydrochloride; ^3^ paracetamol).

The DSC curves of pharmaceutical products containing ibuprofen and additionally pseudoephedrine hydrochloride, phenylephrine hydrochloride, or paracetamol are shown in Figure 2. To interpret these curves, ibuprofen standard and paracetamol standard DSC curves are also presented in this figure. The other ingredients are in the products in small amounts. According to literature data, the melting point of pseudoephedrine hydrochloride is observed in the range of 182–185 °C, while for phenylephrine hydrochloride it is approximately 145 °C [30,31]. The predicted and measured heat of melting values for ibuprofen in products are summarized in Table 1. The DSC curves of all products in this group show a melting peak of ibuprofen with onset at a lower temperature than the reference ibuprofen. The onset temperature for these peaks was in the range of 68.4–73.5 °C. The heat of melting measured for most products is 70.4–98.9% of the predicted heat of melting. The exception in this group is Nurofen Zatoki, for which this value is only 36.8% of the expected value.

On the DSC curves of products containing ibuprofen and pseudoephedrine hydrochloride, no peak characteristic of the second active substance was observed at approximately 182–185 °C [30]. The additional peaks observed for Metafen Zatoki, Modafen Extra Grip, and Nurofen Zatoki are possibly due to excipients.

For products containing ibuprofen and paracetamol (APAP intense, Metafen), the melting peak of ibuprofen on the DSC curve was observed with onset at around 72 °C, so the shift in the melting peak was insignificant for these products. These curves also show the broadened melting peak of paracetamol. The onset of paracetamol melting is shifted by approximately 25–30 °C on these curves compared to the reference paracetamol.

#### 3.1.2. FTIR Results

**Figure 3:**
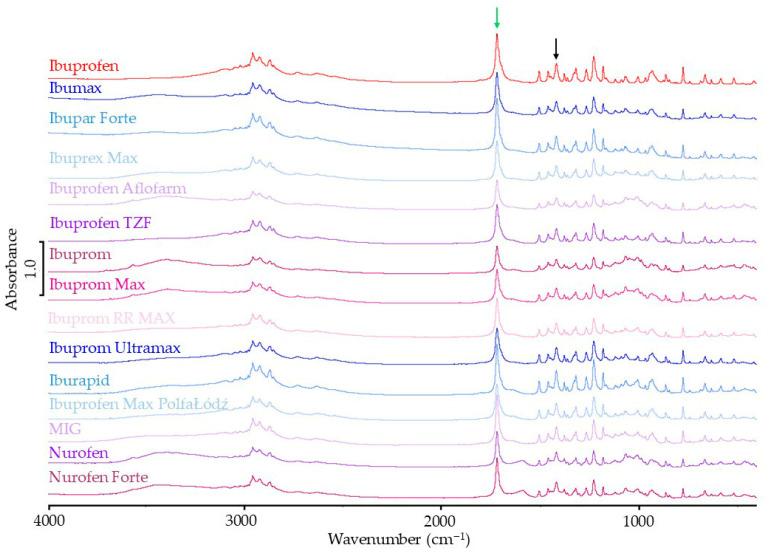
FTIR spectra of ibuprofen standard and pharmaceutical products with ibuprofen.

FTIR spectra of ibuprofen standard and pharmaceutical products containing ibuprofen as the only active ingredient are shown in Figure 3. All bands characteristic of the API were observed in the product spectra. No new bands or disappearance of bands specific to ibuprofen were observed.

To interpret the spectra correctly, their band intensities were standardized. For this purpose, a strong band at 1420 cm^−1^ in the FTIR spectrum of ibuprofen was selected [29,32]. This band is indicated by a black arrow in Figure 3. The intensity of this band does not depend on the polymorphic form of ibuprofen. Regarding this band, the intensity of the other bands has been standardized. Spectrum standardization removes the influence of the active ingredient content in a pharmaceutical product on the result of the analysis of API crystallinity in the product. In the FTIR spectrum, the band at 1721 cm^−1^ is characteristic of the crystalline form of ibuprofen [32]. This band is marked with a green arrow.

The standardized intensity of this band for the ibuprofen standard and ibuprofen products is shown in Table 2. Based on the standardized band intensity, the percentage of the measured value for the products corresponding to the expected band intensity for crystalline ibuprofen was calculated. These values are also presented in Table 2. For most products, these values were lower than the value measured for the API crystalline standard and ranged from 73.7 to 96.3%. The exceptions in this group are Ibupar forte and MIG, for which the intensity of the analyzed band is 99.5 and 102.8% of the expected value, respectively.

**Figure 4:**
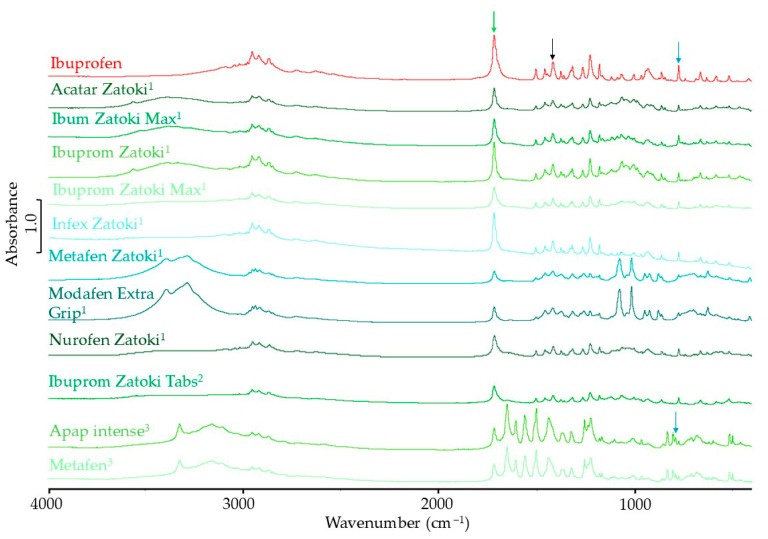
FTIR spectra of ibuprofen standard, paracetamol standard, and pharmaceutical products with ibuprofen and the second API (^1^ pseudoephedrine hydrochloride; ^2^ phenylephrine hydrochloride; ^3^ paracetamol).

The FTIR spectra of products containing ibuprofen and a second API are shown in Figure 4. In the FTIR spectrum of pseudoephedrine hydrochloride and phenylephrine hydrochloride, according to literature data, there are no bands at 1420 cm^−1^ or 1721 cm^−1^ [33,34]. The characteristic bands are marked in Figure 4 with black and green arrows. The standardization process and calculations for products containing ibuprofen and pseudoephedrine hydrochloride or phenylephrine hydrochloride were performed in the same way as for single ibuprofen products.

There is a very strong band in the paracetamol spectrum at around 1437 cm^−1^, which makes standardization of the spectrum against the 1420 cm^−1^ band unreliable for products containing both ibuprofen and paracetamol [35]. For this reason, the standardization of band intensity in FTIR spectra of products containing these two substances was performed with relation to the band at 780 cm^−1^. This band is marked in Figure 4 with a blue arrow in the ibuprofen spectrum and above the spectra of products containing phenylephrine hydrochloride and paracetamol. However, there is no band at 1721 cm^−1^ in the paracetamol spectrum, so the band at this wavenumber could be used in subsequent calculations. For products containing ibuprofen and pseudoephedrine hydrochloride, the standardized band intensity characteristic of the crystalline form of ibuprofen was 48.4–80.6% of the expected value. For the product containing phenylephrine hydrochloride, this value was 69.1%, and for both products containing paracetamol, it was 70.5%.

#### 3.1.3. Raman Spectroscopy Results

**Figure 5:**
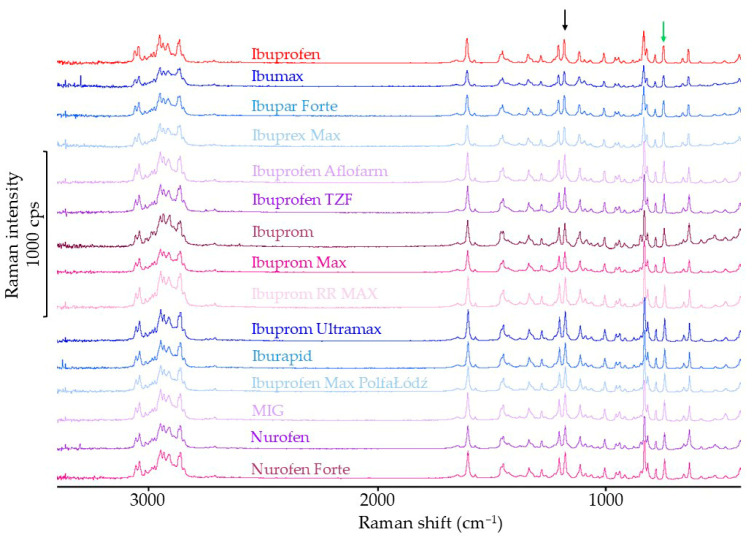
Raman spectra of ibuprofen standard and pharmaceutical products with ibuprofen.

The Raman spectra of ibuprofen and products containing ibuprofen as the single API are shown in Figure 5. The intensity of the band at a shift of 1113 cm^−1^ is independent of the ibuprofen polymorphic form [36]. Therefore, the intensity of the other bands in the Raman spectra of ibuprofen and products containing this substance was standardized. The crystalline form of ibuprofen is confirmed by a band at a shift of 783 cm^−1^, so the normalized intensity of this band was used for subsequent calculations. The reference band in Figure 5 is marked with a black arrow, and the analyzed band is marked with a green arrow. The standardized intensity of this band in pharmaceutical products containing only ibuprofen was 63.7–95.5% of the value obtained for the ibuprofen standard.

**Figure 6:**
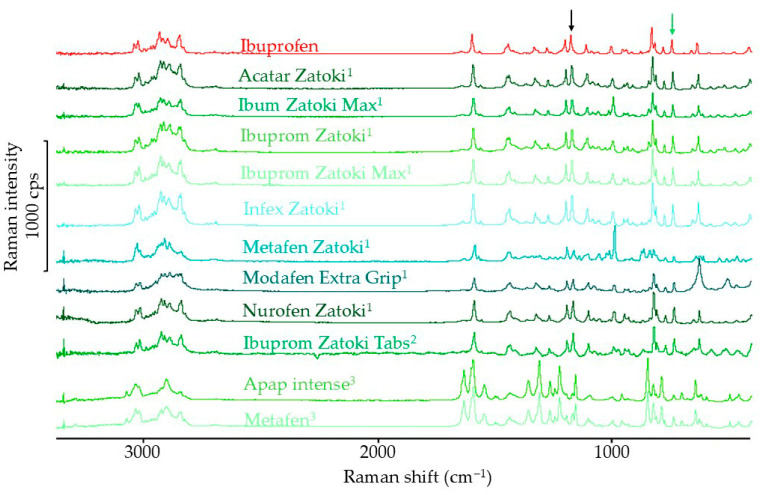
Raman spectra of ibuprofen standard, paracetamol standard, and pharmaceutical products with ibuprofen and with the second API (^1^ pseudoephedrine hydrochloride; ^2^ phenylephrine hydrochloride; ^3^ paracetamol).

The Raman spectra of products containing ibuprofen and a second active ingredient are shown in Figure 6. In the Raman spectrum of pseudoephedrine hydrochloride, there are no bands characteristic of this substance in the regions of 783 cm^−1^ or 1113 cm^−1^ [37]. For this reason, the standardization of the spectra of products containing ibuprofen and pseudoephedrine hydrochloride was carried out in the same way as for products containing ibuprofen only. The characteristic bands are marked with arrows in Figure 6. The standardized crystal band intensity of ibuprofen in the spectra of products containing ibuprofen and pseudoephedrine hydrochloride was 59.7–91.4% of the value calculated for the ibuprofen standard.

In the Raman spectrum of paracetamol and phenylephrine hydrochloride, it was not possible to select bands in such a way that the bands characteristic of ibuprofen did not overlap with the bands of these active substances [38]. Therefore, it was not possible to analyze the characteristic band intensity of the crystalline form of ibuprofen in the Raman spectra of products containing both ibuprofen and phenylephrine hydrochloride or paracetamol.

#### 3.1.4. XRD Results

**Figure 7:**
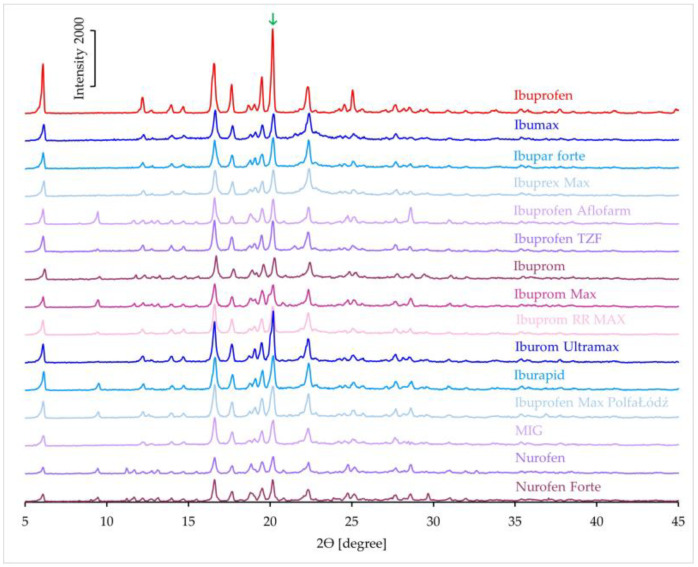
XRD patterns of ibuprofen standard and pharmaceutical products with ibuprofen.

Figure 7 shows the XRD patterns of ibuprofen and products containing ibuprofen as the only active ingredient. The high-intensity peaks in the XRD pattern of ibuprofen clearly indicate its crystalline nature. The XRD patterns of all products also exhibit intense peaks confirming the presence of the crystalline form of the API. An analysis was performed on the intensity of the peak at 20.2° 2θ in the product samples. This peak is marked with a green arrow in Figure 7. Based on the intensity of this peak for pure ibuprofen, its expected intensity in the pattern of the products was calculated. These calculations used the API content (%) in each product (Table 1). Subsequently, the percentage of the measured peak intensity relative to the predicted value was determined. The results of this analysis are presented in Table 3, with values ranging from 43.8 to 78.6%.

**Figure 8:**
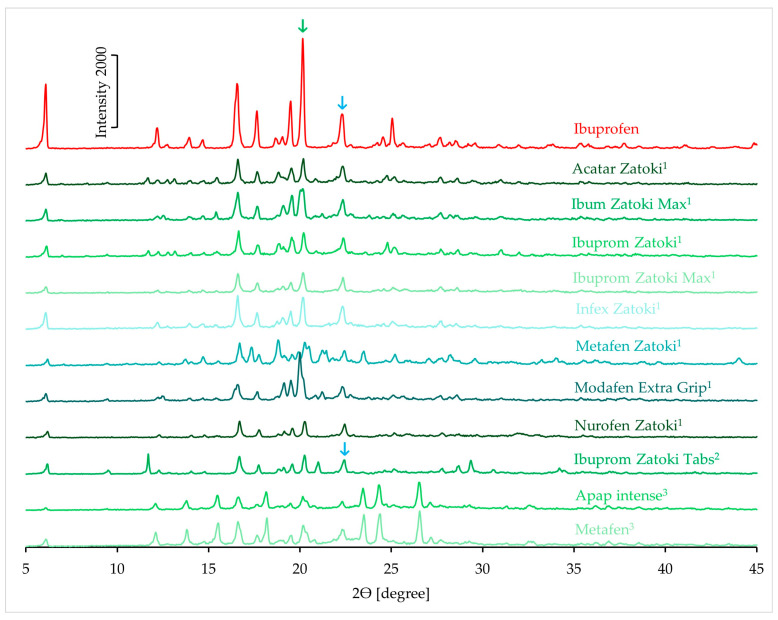
XRD patterns of ibuprofen standard and pharmaceutical products with ibuprofen and the second API (^1^ pseudoephedrine hydrochloride; ^2^ phenylephrine hydrochloride; ^3^ paracetamol).

The XRD patterns of products containing ibuprofen and the second active ingredient are shown in Figure 8. The appearance of characteristic peaks in the spectra confirms that the products contain crystalline ibuprofen. In the XRD pattern of pseudoephedrine hydrochloride, there is no peak at approximately 20° 2θ, therefore, the crystallinity analysis of products containing this active substance was performed on the peak at 20.2° [39]. This peak is indicated with a green arrow in Figure 8. The calculated values for these products are summarized in Table 3. The intensity of the analyzed peak in these products ranges from 27.9% to 75.3% of the predicted value.

According to the literature, the XRD patterns of phenylephrine hydrochloride and paracetamol exhibit peaks at around 20° 2θ, but no peak is observed at 22° 2θ [40,41]. Therefore, the analysis of ibuprofen crystallinity in these spectra was based on the peak at 22° 2θ. This peak is indicated by a blue arrow in Figure 8. The values calculated for these products are presented in Table 3. For the product containing phenylephrine hydrochloride, the intensity of this peak is 95.6% of the predicted value. In products containing paracetamol, these values exceed the predicted value several times over. The intensity of these peaks is likely also influenced by other ingredients present in the products.

### 3.2. Naproxen Products

#### 3.2.1. DSC Results

**Figure 9:**
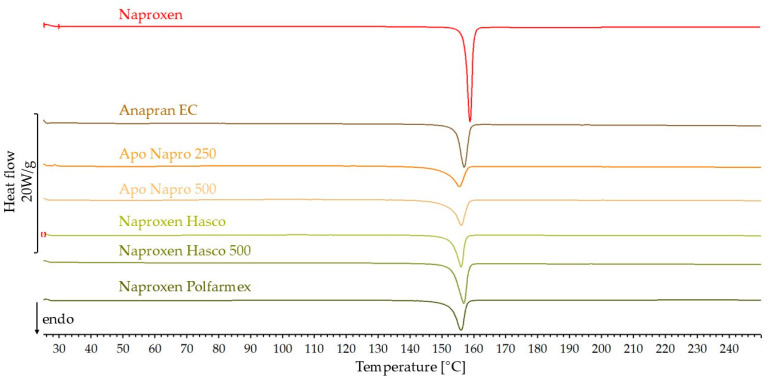
DSC curves of naproxen standard and pharmaceutical products with naproxen.

The DSC curves for naproxen and products containing this substance are shown in Figure 9. Table 1 summarizes the onset temperature of the naproxen melting peak, the peak temperature, and heat of melting. In addition, the API content in the product and the percentage of predicted heat of melting obtained for each product are presented. On the DSC curve of each naproxen product, the melting peak is slightly shifted toward lower temperatures and slightly broadened. The measured heat of melting represents 57.1–81.3% of the predicted value. No peaks corresponding to excipients were observed on the DSC curves of these products.

#### 3.2.2. FTIR Results

**Figure 10:**
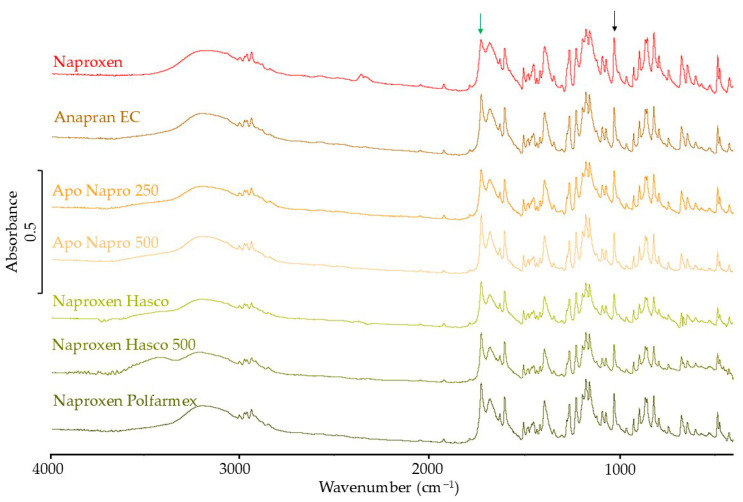
FTIR spectra of naproxen standard and pharmaceutical products with naproxen.

Figure 10 shows the FTIR spectrum of the naproxen standard and products containing this substance. According to the literature data, the intensity of the 1020 cm^−1^ band in the FTIR spectrum of naproxen does not depend on the polymorphic form [42]. This band is marked with a black arrow in Figure 10. The standardization of the intensity of the bands in the spectrum of naproxen and products containing this active substance was therefore carried out using this band. The existence of the crystalline form of naproxen is confirmed by the band at 1728 cm^−1^. This band is marked with a green arrow. The standardized band intensity values and the percentage of the expected band intensity calculated based on the naproxen reference spectrum are presented in Table 4. These results ranged from 72.9% to 94.3% of the predicted value.

#### 3.2.3. Raman Spectroscopy Results

**Figure 11:**
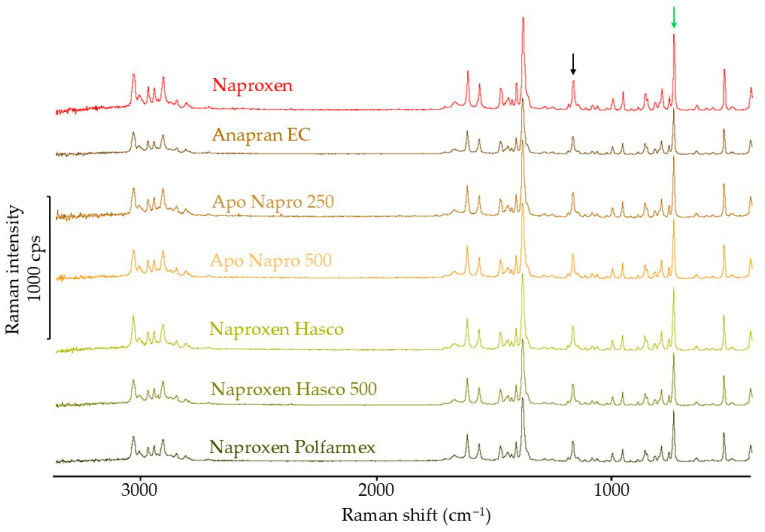
Raman spectra of naproxen standard and pharmaceutical products with naproxen.

The Raman spectra of the naproxen standard and products containing this substance are shown in Figure 11. In the Raman spectrum of naproxen, the band at 1170 cm^−1^ is independent of the crystal form, and this band was used as a reference for standardizing band intensity [43]. Confirmation of the crystal form is possible based on the band at 760 cm^−1^. Therefore, the intensity of this band was used for subsequent calculations. The characteristic bands are marked with black and green arrows in Figure 11. The standardized band intensity of 760 cm^−1^ in naproxen products represents 93.1–98.2% of the value expected based on the naproxen reference spectrum. Table 4 summarizes the values calculated based on the Raman spectra of naproxen and naproxen products.

#### 3.2.4. XRD Results

**Figure 12:**
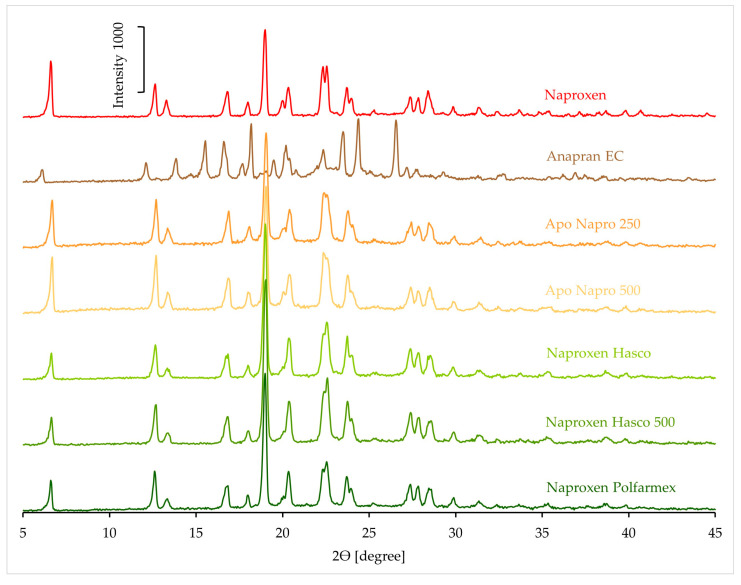
XRD patterns of naproxen standard and pharmaceutical products with naproxen.

The XRD pattern of naproxen and products containing this substance is shown in Figure 12. High-intensity peaks indicate crystalline forms in the case of the substance and products. In most cases, the intensity of the peaks in the product spectra is higher than that of the naproxen standard, which is difficult to explain. Therefore, no further calculations of peak intensities in product spectra were performed. An exception in this group is Anapran EC, whose XRD spectrum shows peaks confirming its crystalline form, but the position of these peaks differs from that of the reference naproxen.

### 3.3. Naproxen Sodium Products

#### 3.3.1. DSC Results

**Figure 13:**
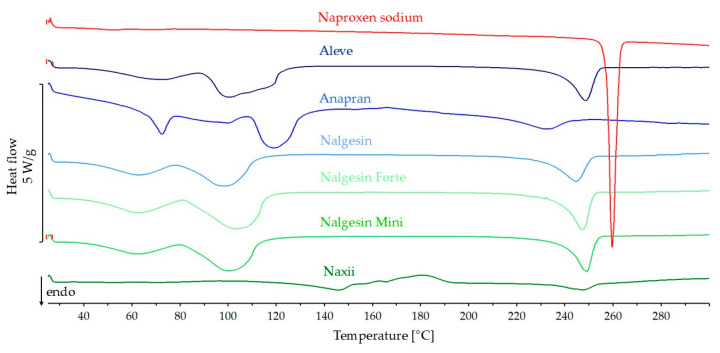
DSC curves of naproxen sodium standard and pharmaceutical products with naproxen sodium.

Figure 13 shows the DSC curves for naproxen sodium standard and products containing this substance. The API content calculated based on the dose is 62.5–69.7% of the mass of the pharmaceutical product with naproxen sodium. The values describing the API melting point in products are listed in Table 1. On the DSC curves of the products, the melting peak of naproxen sodium was strongly broadened and shifted towards lower temperatures. Additional signals from excipients were observed in all DSC curves of the products. The heat of melting of naproxen sodium in Naxii and Anapran products is only 17.6% and 45.3% of the predicted value, respectively. For other products, this value ranged from 65.6% to 79.5%.

#### 3.3.2. FTIR Results

**Figure 14:**
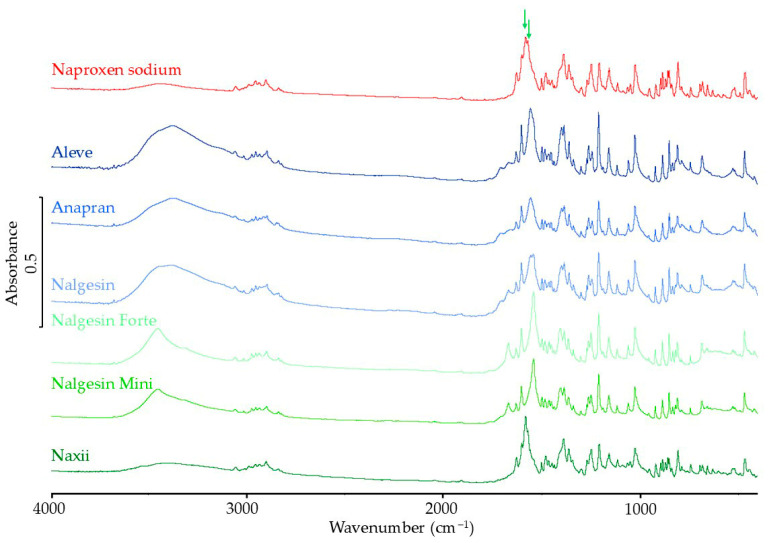
FTIR spectra of naproxen sodium standard and pharmaceutical products with naproxen sodium.

FTIR spectra of the naproxen sodium standard and products containing this substance are shown in Figure 14. According to the literature, the crystalline form is confirmed by bands at 1574–1583 cm^−1^ [42,43]. These characteristic bands are indicated by arrows in Figure 14. In the spectrum of the naproxen sodium standard, these bands are clearly marked, but in the spectra of the products in this range, several bands are visible, usually shifted in relation to the standard spectrum. Due to differences in the shape and position of the absorption bands of naproxen sodium in the products compared to the reference API, it was not possible to analyze the band heights indicating the presence of the crystalline form of naproxen sodium. Considering the complicated composition of the tablet matrix and the possible influence of excipients on the polymorphism of naproxen sodium in products, it is difficult to determine whether this influence is related to the overlapping of signals from excipients or the amorphization of naproxen sodium.

#### 3.3.3. Raman Spectroscopy Results

**Figure 15:**
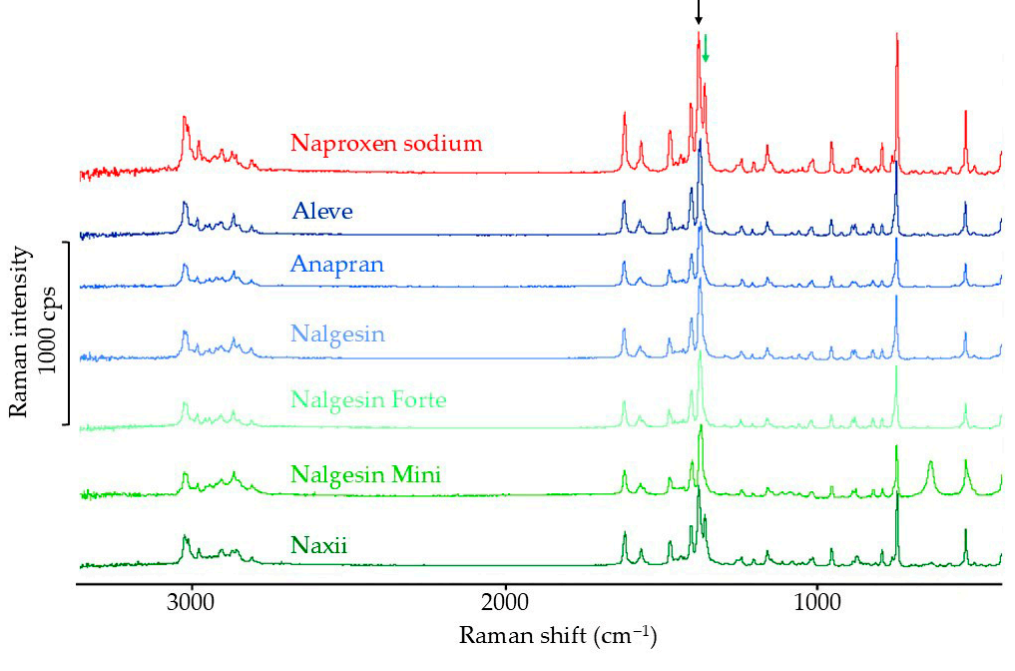
Raman spectra of naproxen sodium standard and pharmaceutical products with naproxen sodium.

According to the literature, the crystalline form of naproxen sodium in the Raman spectrum is confirmed by a band at 1371 cm^−1^ [43]. The locations of both bands are indicated by arrows in Figure 15. The lack of this band indicates an amorphous API form. The band at 1371 cm^−1^ was observed only in the spectrum of the naproxen sodium standard and in the Naxii product (Figure 15). This band is close to the band at 1390 cm^−1^, which is independent of the polymorphic form of this substance. In the spectra of the other products, a single band was observed in the range of 1376–1383 cm^−1^. Due to the absence of the band characteristic of crystalline naproxen sodium in the Raman spectra of products containing this API, it was not possible to further interpret the band intensities. The presence of broad band products containing naproxen sodium in the Raman spectra, in the range where the characteristic band for the crystalline form was expected, significantly complicates the interpretation of the results. There is no clear answer as to whether this phenomenon results from the presence of excipients or clearly proves the amorphization of naproxen sodium in the products.

#### 3.3.4. XRD Results

**Figure 16:**
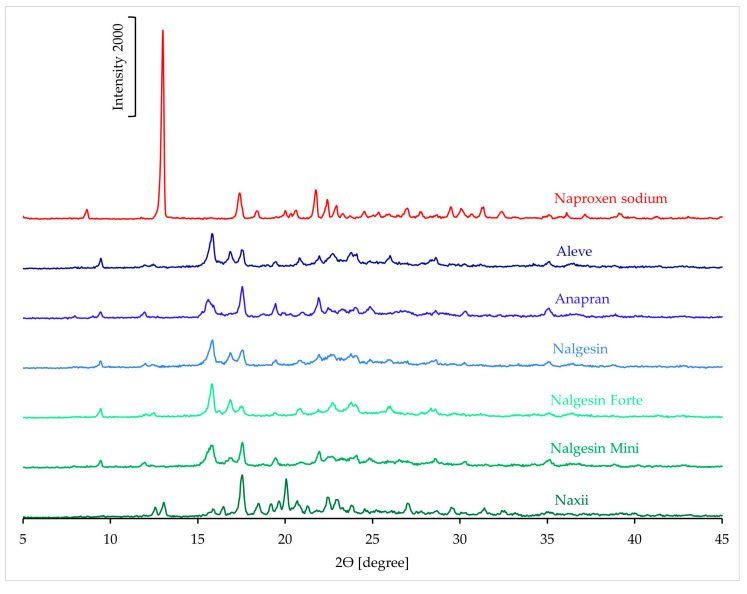
XRD pattern of naproxen standard and pharmaceutical products with naproxen sodium.

Figure 16 shows the XRD patterns of naproxen sodium and products containing this active ingredient. The pattern of pure naproxen sodium shows a characteristic, very intense peak at 13° 2θ. This peak is not observed in the spectra of most products. However, the Naxii product is an exception, as a small signal is observed in its XRD pattern at this position. Additionally, the spectra of most products exhibit an intense peak at 15.8° 2θ, which is not present in the reference pattern of naproxen sodium.

## 4. Discussion

If there are no physical interactions between the components of a pharmaceutical product, the melting point of the crystalline active substance should be consistent with the reference value for the standard API [44]. This compatibility applies to the onset of API melting as well as the heat of melting.

The clearly visible melting point of ibuprofen in pharmaceutical products confirms that the products contain the crystalline form of the API. This applies to all products containing ibuprofen, regardless of the presence of a second API. However, the reduction in the heat of melting and the shift of the melting peak to lower temperatures compared to the reference ibuprofen confirm a partial reduction in the crystalline form of ibuprofen in the products. The lack of a melting peak for pseudoephedrine hydrochloride and phenylephrine hydrochloride is probably due to the small amounts of these substances in pharmaceutical products. The pseudoephedrine hydrochloride content ranges from 4.3 to 8.2% of the tablet weight, while the phenylephrine hydrochloride content is 1.5%.

The position of the melting peak on the DSC curve may also be influenced by factors such as the formation of eutectic mixtures or the formation of solid solutions [45]. For this reason, it is important to confirm conclusions regarding the reduction of API crystallinity using FTIR and Raman spectroscopic methods. Analysis of band intensities in FTIR and Raman spectra of ibuprofen products confirmed that interactions between components in the products lead to a reduction in API crystallinity. This effect was noticed in products with ibuprofen as the only API and in products with pseudoephedrine hydrochloride as an extra ingredient. Signals from phenylephrine hydrochloride or paracetamol in the Raman spectra overlapped with the bands of ibuprofen. This made it impossible to confirm the effect of the tablet matrix on the crystallinity of ibuprofen in these products using this technique. The results indicate that the reduction in crystallinity in products with an added API is more pronounced than in preparations containing only ibuprofen. The results obtained during XRD testing also confirmed a partial reduction in the crystalline form in pharmaceutical products containing ibuprofen, regardless of whether this substance was the only API or whether the product contained an additional active substance.

The DSC results also indicate the crystalline form of naproxen in products containing this substance. At the same time, the shift of the melting peak towards lower temperatures and the lower-than-expected heat of melting confirm a partial reduction in the crystallinity of naproxen in products. Similar results were obtained using FTIR. The results obtained using Raman spectroscopy indicate only a slight effect of the tablet matrix on the crystallinity of naproxen in pharmaceutical products. In the XRD results for naproxen products, due to very intense signals, it was not possible to confirm the results obtained by other methods.

In naproxen sodium products, DSC results indicate a significant reduction in API crystallinity. In the FTIR and Raman spectra of most products, it was not possible to identify bands characteristic of the crystalline form, which confirms the possibility of amorphization of naproxen sodium under the influence of excipients in solid pharmaceutical products containing this substance. XRD results confirm a significant reduction or the possibility of complete amorphization of naproxen sodium in pharmaceutical products.

## 5. Conclusions

The DSC, FRIR, Raman, and XRD results show that pharmaceutical products with ibuprofen, naproxen, and naproxen sodium contain APIs in crystalline form, but excipients lead to a reduction in the crystallinity of these substances. The results are only a preliminary analysis aimed at assessing whether and to what extent the crystallinity of APIs in pharmaceutical products can be reduced by excipients. However, the research needs to be continued and expanded, e.g., with NMR (Nuclear Magnetic Resonance) in the solid state. Results indicate the need for extensive research to determine the effect of the matrix in solid pharmaceutical formulations on API crystallinity. Further studies are needed to clarify the effect of individual excipients on API polymorphism by performing excipient-controlled experiments with using model formulations because individual excipients were not systematically varied or tested in isolation. However, analyses correlating the presence or absence of specific excipients in the product with the crystallinity of the API in the pharmaceutical product are equally important. This is particularly important due to differences in solubility and bioavailability between various polymorphic and amorphous forms of API. Such research enables the expansion of knowledge in the field of the proper selection of excipients for solid pharmaceutical formulations.

## Figures and Tables

**Table 1:** API content in a pharmaceutical product, predicted and measured heat of melting, and values describing the API melting peak on the DSC curve: onset—initiation peak temperature, peak—maximum peak temperature.

Ibuprofen						
Product Name	API Content in Product [%]	Heat of Melting [J/g]		Percentage of Predicted Heat of Melting	Onset [°C]	Peak [°C]
		Predicted	Measured			
Ibuprofen			–128.1		74.7	77.0
Ibumax	54.6	−69.9	−54.2	77.5	69.5	73.7
Ibupar forte	62.3	−79.8	−72.6	91.0	69.5	74.5
Ibuprex Max	56.2	−72.0	−66.7	92.6	72.6	75.4
Ibuprofen Aflofarm	44.0	−56.3	−61.2	108.8	73.5	76.2
Ibuprofen TZF	67.9	−86.9	−62.3	71.6	68.1	72.3
Ibuprom	39.4	−50.5	−42.9	85.1	71.9	75.0
Ibuprom Max	46.7	−59.8	−53.0	88.6	71.4	75.1
Ibuprom RR MAX	59.5	−76.2	−62.6	82.2	72.4	75.0
Ibuprom Ultramax	77.3	−99.0	−81.3	82.2	71.7	75.7
Iburapid	71.6	−91.6	−78.3	85.4	74.0	76.0
Ibuprofen Max PolfaŁódź	71.8	−91.9	−73.3	79.8	74.3	76.5
MIG	60.3	−77.3	−69.1	89.5	71.2	74.6
Nurofen	46.9	−60.0	−50.6	84.4	72.4	75.5
Nurofen Forte	46.6	−59.6	−69.5	116.6	72.1	74.9
**Ibuprofen with different API**						
Acatar Zatoki ^1^	37.5	−48.0	−39.3	81.8	70.2	74.5
Ibum Zatoki Max ^1^	48.6	−62.3	−50.2	80.6	71.9	75.5
Ibuprom Zatoki ^1^	37.7	−48.3	−43.3	89.6	70.2	74.0
Ibuprom Zatoki Max ^1^	54.2	−69.5	−60.5	87.1	69.5	73.8
Infex Zatoki ^1^	53.3	−68.3	−65.3	95.6	68.4	73.7
Metafen Zatoki ^1^	28.5	−36.5	−25.7	70.4	70.2	74.8
Modafen Extra Grip ^1^	34.4	−44.1	−34.9	79.3	67.2	72.3
Nurofen Zatoki ^1^	54.6	−70.0	−25.7	36.8	70.2	74.8
Ibuprom Zatoki Tabs ^2^	48.6	−62.2	−61.6	98.9	73.5	76.1
APAP intense ^3^	20.8	−26.6	−20.0	75.2	72.1	74.8
Metafen ^3^	28.4	−36.3	−27.4	75.4	72.3	75.5
**Naproxen**						
Naproxen			–170.9		156.6	157.1
Anapran EC	82.7	–141.4	–114.9	81.3	153.9	155.6
Apo Napro 250	81.0	–138.4	−90.8	65.6	150.9	154.7
Apo Napro 500	79.9	–136.6	–102.9	75.3	152.3	155.3
Naproxen Hasco	92.5	–158.1	−90.4	57.1	152.6	155.0
Naproxen Hasco 500	91.7	–156.8	–122.6	78.2	152.7	155.7
Naproxen Polfarmex	89.8	–153.5	−99.8	65.0	152.4	155.1
**Naproxen sodium**						
Naproxen sodium			–136.9		256.8	257.9
Aleve	69.4	−95.0	−62.3	65.6	239.2	248.4
Anapran	67.8	−92.7	−42.0	45.3	213.6	231.4
Nalgesin	62.5	−85.6	−59.7	69.8	231.2	244.5
Nalgesin Forte	69.7	−95.4	−75.8	79.5	238.4	247.0
Nalgesin Mini	68.1	−93.2	−64.8	69.5	240.6	248.9
Naxii	67.3	−92.1	–16.2	17.6	235.3	246.4

As the second active ingredient: ^1^ pseudoephedrine hydrochloride; ^2^ phenylephrine hydrochloride; ^3^ paracetamol.

**Table 2:** Standardized intensity of bands and percentage of reference intensity of band in FTIR and Raman spectra of ibuprofen and its products.

	FTIR Spectra		Raman Spectra	
Sample Name	Standardized Intensity of Band 1721 cm^−1^	Percentage of Reference Intensity of Band in Spectra	Standardized Intensity of Band 1113 cm^−1^	Percentage of Reference Intensity of Band in Spectra
Ibuprofen	2.17	-	0.78	-
Ibumax	2.08	95.9	0.50	63.7
Ibupar forte	2.16	99.5	0.62	79.1
Ibuprex Max	2.06	94.9	0.58	73.9
Ibuprofen Aflofarm	1.81	83.4	0.72	92.4
Ibuprofen TZF	2.09	96.3	0.73	93.4
Ibuprom	1.64	75.6	0.58	75.0
Ibuprom Max	1.81	83.4	0.65	83.6
Ibuprom RR MAX	2.11	97.2	0.60	76.9
Ibuprom Ultramax	1.73	79.7	0.74	95.5
Iburapid	1.80	82.9	0.74	94.8
Ibuprofen Max PolfaŁódź	1.60	73.7	0.70	89.9
MIG	2.23	102.8	0.65	83.5
Nurofen	1.89	87.1	0.62	78.9
Nurofen Forte	1.97	90.8	0.65	83.2
**Ibuprofen with different API**				
Acatar Zatoki ^1^	1.55	71.4	0.52	66.9
Ibum Zatoki Max ^1^	1.64	75.6	0.64	82.4
Ibuprom Zatoki ^1^	1.75	80.6	0.52	67.2
Ibuprom Zatoki Max ^1^	1.59	73.3	0.71	91.4
Infex Zatoki ^1^	1.59	73.3	0.66	84.0
Metafen Zatoki ^1^	1.11	51.2	0.52	67.0
Modafen Extra Grip ^1^	1.05	48.4	0.47	59.7
Nurofen Zatoki ^1^	1.56	71.9	0.63	80.7
Ibuprom Zatoki Tabs ^2^	1.50	69.1	no	no
Ibuprofen (stardadized to 780 cm^−1^)	2.54	-		
APAP intense ^3^	1.79	70.5	no	no
Metafen ^3^	1.79	70.5	no	no

no-not obtained; as the second active ingredient: ^1^ pseudoephedrine hydrochloride; ^2^ phenylephrine hydrochloride; ^3^ paracetamol.

**Table 3:** Peak intensities and percentage of reference peak intensity in the XRD patterns of ibuprofen and its products.

Product Name	Intensity of XRD Peak 20.2 (2θ [Degree])		Percentage of Predicted Peak Intensity
	Predicted	Measured	
Ibuprofen		3068	
Ibumax	1675	1004	59.9
Ibupar Forte	1911	1134	59.3
Ibuprex Max	1724	988	57.3
Ibuprofen Aflofarm	1350	918	68.0
Ibuprofen TZF	2083	1136	54.4
Ibuprom	1209	807	66.8
Ibuprom Max	1433	827	57.7
Ibuprom RR MAX	1825	1050	57.5
Ibuprom Ultramax	2372	1863	78.6
Iburapid	2197	1274	58.0
Ibuprofen Max PolfaŁódź	2203	1173	53.2
MIG	1850	944	51.0
Nurofen	1439	630	43.8
Nurofen Forte	1430	803	56.2
Acatar Zatoki ^1^	1151	735	63.9
Ibum Zatoki Max ^1^	1491	913	61.2
Ibuprom Zatoki ^1^	1157	690	59.7
Ibuprom Zatoki Max ^1^	1663	582	35.0
Infex Zatoki ^1^	1635	899	55.0
Metafen Zatoki ^1^	874	658	75.3
Modafen Extra Grip ^1^	1055	685	64.9
Nurofen Zatoki ^1^	1675	468	27.9
**Intensity of XRD peak 22 (2θ [degree])**			
Ibuprofen		183	
Ibuprom Zatoki Tabs ^2^	89	85	95.6
APAP intense ^3^	38	99	260.1
Metafen ^3^	52	222	427.2

As the second active ingredient: ^1^ pseudoephedrine hydrochloride; ^2^ phenylephrine hydrochloride; ^3^ paracetamol.

**Table 4:** Standardized intensity of bands and percentage of reference intensity of band in FTIR and Raman spectra of naproxen and its products.

	FTIR Spectra		Raman Spectra	
	Standardized Intensity of Band 1728 cm^−1^	Percentage of Reference Intensity of Band in Spectra	Standardized Intensity of Band 760 cm^−1^	Percentage of Reference Intensity of Band in Spectra
Naproxen	1.40	-	0.46	-
Anapran EC	1.17	83.6	0.45	97.3
Apo Napro 250	1.02	72.9	0.43	93.2
Apo Napro 500	1.32	94.3	0.43	93.1
Naproxen Hasco	1.16	82.9	0.44	96.3
Naproxen Hasco 500	1.09	77.9	0.45	98.2
Naproxen Polfarmex	1.09	77.9	0.45	97.5

## Data Availability

Data is contained within the article.

## Supplementary Materials

The following supporting information can be downloaded at: https://www.mdpi.com/article/10.3390/mps8050099/s1, Table S1: Analyzed products manufacturer and serial number.
