# Supplementary material for: Analysis of the Effect of the Tablet Matrix on the Polymorphism of Ibuprofen, Naproxen, and Naproxen Sodium in Commercially Available Pharmaceutical Formulations
Source: Methods Protoc. 2025 Sep 1;8(5):99. doi: 10.3390/mps8050099 (PMC12452562; doi:10.3390/mps8050099)
Supplement: Supplementary file 1 [file mps-08-00099-s001.zip › mps-3754083-supplementary.pdf]

SUPPLEMENTARY MATERIAL

Analysis of the Effect of the Tablet Matrix on the Polymorphism of Ibuprofen, Naproxen, and Naproxen Sodium in Commercially Available Pharmaceutical Formulations

Edyta Leyk[1], Marcin Środa[2], Gracjan Maślanka[1], Patrycja Nowaczyk[1], Amelia Orzołek[1], Hanna Grodzka[1], Aleksandra Kurek[1], Olaf Knut[1], Julia Michalak[1], Jonatan Płachciak[1], Alina Plenis[1]*

[1] Department of Analytical Chemistry, Faculty of Pharmacy, Medical University of Gdansk, Gen. J. Hallera 107, 80-416 Gdansk, Poland; edyta.leyk@gumed.edu.pl (E.L.), gmaslanka@gumed.edu.pl (G.M), p.nowaczyk@gumed.edu.pl (P.N), ameliaorzolek@gumed.edu.pl (A.O.), hannagrodzka@gumed.edu.pl (H.G.), aleksandra.kurek@gumed.edu.pl (A.K.), olfinx@gumed.edu.pl (O.K.), juliamichalak1234567@gumed.edu.pl (J.M.), jonatan.plachciak@gumed.edu.pl (J.P.), aplenis@gumed.edu.pl (A.P.).

[2] Faculty of Materials Science and Ceramics, AGH University of Science and Technology, A. Mickiewicza 30, 30-059 Kraków, Poland, msroda@agh.edu.pl (M.Ś.)

* Correspondence: aplenis@gumed.edu.pl (A.P.); Tel.: +48-58-3491096; Fax: +48-58-349152

Table S1. Analyzed products manufacturer and serial number.

Active pharmaceutical ingredient/s	Product name	Manufacturer
Ibuprofen	Ibumax	Vitabalans Oy, Finland
	Ibupar forte	Adamed Pharma S.A., Poland
	Ibuprex Max	Olimp Laboratories Sp. z o.o., Poland
	Ibuprofen Aflofarm	Aflofarm Farmacja Polska Sp. z o.o., Poland
	Ibuprofen TZF	Tarchomińskie Zakłady Farmaceutyczne „Polfa" S.A., Poland
	Ibuprom	US Pharmacia Sp. z o.o., Poland
	Ibuprom Max	US Pharmacia Sp. z o.o., Poland
	Ibuprom RR MAX	US Pharmacia Sp. z o.o., Poland
	Ibuprom Ultramax	US Pharmacia Sp. z o.o., Poland
	Iburapid	Wörwag Pharma Operations Sp. z o.o., Poland
	Ibuprofen Max PolfaŁódź	Laboratoria Polfa Łódź, Poland
	MIG	Berlin-Chemie AG, Germany
	Nurofen	Reckitt Benckiser Healthcare International Ltd, UK
	Nurofen Forte	Reckitt Benckiser Healthcare International Ltd, UK
Ibuprofen and pseudoephedrine hydrochloride	Acatar Zatoki	US Pharmacia Sp. z o.o., Poland
	Ibum Zatoki Max	HASCO-LEK S.A., Poland
	Ibuprom Zatoki	US Pharmacia Sp. z o.o., Poland
	Ibuprom Zatoki Max	US Pharmacia Sp. z o.o., Poland
	Infex Zatoki	Merckle GmbH, Germany
	Metafen Zatoki	Wörwag Pharma Operations Sp. z o.o., Poland
	Modafen Extra Grip	Zentiva k.s., Czech Republic
	Nurofen Zatoki	Reckitt Benckiser Healthcare International Ltd, UK
Ibuprofen and phenylephrine hydrochloride	Ibuprom Zatoki Tabs	US Pharmacia Sp. z o.o., Poland
Ibuprofen and paracetamol	APAP intense	Rontis Hellas Medical and Pharmaceutical Products S.A., Grece
	Metafen	Wörwag Pharma Operations Sp. z o.o., Poland
Naproxen	Anapran EC	Adamed Pharma S.A.
	Apo Napro 250	APL Swift Services, Malta
	Apo Napro 500	APL Swift Services, Malta
	Naproxen Hasco	HASCO-LEK S.A., Poland
	Naproxen Hasco 500	HASCO-LEK S.A., Poland
	Naproxen Polfarmex	Polfarmex S.A., Poland
Naproxen sodium	Aleve	Bayer Bitterfeld GmbH, Germany
	Anapran	Adamed Pharma S.A., Poland
	Nalgesin	KRKA, d.d., Slovenia
	Nalgesin Forte	KRKA, d.d., Slovenia
	Nalgesin Mini	KRKA, d.d., Slovenia
	Naxii	US Pharmacia Sp. z o.o., Poland
